# A New Adaptation of the Face Mask in Control of Contagious Disease

**Published:** 1918-11

**Authors:** 


***A New Adaptation of the Face Mask in Control of Contagious Disease.*** By Joseph A. Capps. *Journal of the American Medical Association*, March 30, 1918.

The contraction of a secondary infection directly from another individual in the same ward demands the most serious effort in the way of prevention.

Most contagious diseases, such as measles, German measles, scarlet fever, pertussis, meningitis and diphtheria, are transmitted from one person to another by means of the secretions of the nose and mouth. Coughing, sneezing, and talking convey the bacteria-laden droplets of mucus through the air. In addition to the cubicle system, the Camp Grant Base Hospital has carried out the practice of requiring physicians, nurses, and ward men to wear gowns and gauze face masks. With all these measures, however, it was impossible to prevent the spread of cross infection from patient to patient, since there were no facilities for effectively separating patients in a ward once exposed to a fresh contagious disease. The cubicle segregates the bed patients, but it is almost impossible to confine convalescent soldiers to bed day and night. Furthermore, it is not possible to furnish the necessary service — the nursing, bathing and feeding — to render such segregation effective.

The face mask has proved an undoubted success in protecting physicians and nurses from contagion. It seems reasonable that patients, by wearing the mask, could receive similar protection from either primary contagion, or, if already ill from one infection, from a second contagion.

The essential principle of the method is that patients out of bed and out of the cubicle must never be unmasked, except when alone in the wash room. In order to make it effective the following rules are posted in each ward and latrine :

(1) A face mask must be worn continuously by all patients when out of cubicle.

(2) In the latrine the mask may be removed only by permission and under the direction of the ward nurse.

(3) Wash basins and bath tub are not to be used. Use only liquid soap from container.

(4) Only one patient will be allowed in wash room at one time.

(5) Masks may be removed when patients are in bed.

(6) Sheets between beds are not to be drawn back.

(7) No smoking allowed.

(8) Corps men, nurses and surgeons should wear masks and gowns when on duty in the ward.

(9) All eating utensils should be sterilized after each meal.

To guard against another danger of cross infection, every patient before entering an ambulance should put on a face mask and continue to wear it until he reaches a bed in the ward. This applies to all patients whether they have any communicable disease or not.

